# Roles of phosphotase 2A in nociceptive signal processing

**DOI:** 10.1186/1744-8069-9-46

**Published:** 2013-09-08

**Authors:** Yun Wang, Yongzhong Lei, Li Fang, Yonggao Mu, Jing Wu, Xuan Zhang

**Affiliations:** 1 Department of Anesthesiology, Beijing Chaoyang Hospital, Capital Medical University, Beijing 100020, China; 2 Department of Neuroscience and Cell Biology, The University of Texas Medical Branch, Galveston, TX 77555-0517, USA; 3 Department of Neurosurgery, Cancer Center, Sun Yat-sen University, Guangzhou 510060, China; 4 Department of Neurosurgery and Neurology, Lineberger Cancer Center, School of Medicine, University of North Carolina, Chapel Hill, NC 27599, USA

## Abstract

Multiple protein kinases affect the responses of dorsal horn neurons through phosphorylation of synaptic receptors and proteins involved in intracellular signal transduction pathways, and the consequences of this modulation may be spinal central sensitization. In contrast, the phosphatases catalyze an opposing reaction of de-phosphorylation, which may also modulate the functions of crucial proteins in signaling nociception. This is an important mechanism in the regulation of intracellular signal transduction pathways in nociceptive neurons. Accumulated evidence has shown that phosphatase 2A (PP2A), a serine/threonine specific phosphatase, is implicated in synaptic plasticity of the central nervous system and central sensitization of nociception. Therefore, targeting protein phosphotase 2A may provide an effective and novel strategy for the treatment of clinical pain. This review will characterize the structure and functional regulation of neuronal PP2A and bring together recent advances on the modulation of PP2A in targeted downstream substrates and relevant multiple nociceptive signaling molecules.

## Introduction

Intracellular signal transduction pathways play a pivotal role in the maintenance of biological processes such as cell growth, proliferation, survival, and metabolism in all cells and tissues. It has been demonstrated that a variety of intracellular signal transduction pathways are involved in the physiological or patho-physiological responses to noxious stimuli [1-3]. The opposing reactions of phosphorylation and de-phosphorylation of critical cellular proteins are decisive to such pathways [4,5]. Protein kinases and phosphatases catalyze protein phosphorylation and de-phosphorylation reactions, respectively. While in the past, much attention has been paid to the regulation of protein kinases, it is now apparent that protein phosphatases are highly regulated enzymes that play an equally important role in the control of protein phosphorylation. Accumulated evidence has shown that protein kinases are widely implicated in pain modulation. Several protein kinases affect the responses of spinal cord dorsal horn neurons through phosphorylation of synaptic receptors and proteins involved in intracellular signal transduction pathways, and the consequences of this modulation can regulate the process of central sensitization [2,6-10]. However, much less is known about the role of their counterparts, protein phosphatases, in nociception. Recent studies have provided evidence that the a member of protein phosphatase family, protein phosphatase 2A (PP2A), is involved in synaptic plasticity in the central nervous system (CNS) or central sensitization of pain, suggesting a new promising molecular target for pain control [11-15].

Serine/threonine specific phosphatase is one of major classes of protein phosphatases that catalyse the de-phosphorylation of serine and threonine residues. According to their biological characteristics, sensitivities to specific inhibitors and substrates, serine/threonine specific phosphatase can be divided into four major subtypes, PP1, PP2A, PP2B and PP2C [16]. Among this family members, PP2C belongs to a separate gene family since it has a distinct structure from the others, whereas PP1, PP2A and PP2B have similar primary amino acid sequences. There are other serine/threonine phosphatases identified as well, including PP4, PP5, PP6 and PP7. Unlike PP1 and PP2A, the *in vitro* basic activities of PP4, PP5, PP6, and PP7 are extremely low. Of these subtypes, PP2A is the most abundant serine/threonine protein phosphatase in mammalian cells and is expressed at higher levels in the CNS [17]. This review will characterize the structure and functional regulations of PP2A and highlight recent advances in the involvement of PP2A in de-phosphorylation of specific downstream substrates and nociceptive signal processing.

### The structure and localization of PP2A

**Figure 1:**
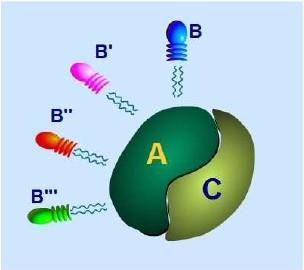
**Structure of protein phosphatase 2A holoenzymes.** A is the structural subunit and scaffolding protein, and C is the catalytic subunit. B/B’/B”/B”’ are the variable and regulatory subunits. PP2A predominantly exists in cells as a heterotrimetic holoenzyme, which consists of a 36 kDa catalytic subunit (PP2A-C), a 65kDa structural subunit (PP2A-A) forming a core enzyme, and a variable regulatory subunit (PP2A-B). The A structure subunit recruits the C catalytic subunit to form the core dimer, which acts as a scaffold for the C and B subunits of the enzyme. Four B subunit families including PR55or B, PR61 or B’, PR72/130 or B” and PR93/PR110 or B”’ interact via the same or overlapping sites within the A subunit of the core dimer. The PP2A holoenzyme's substrate specificity, enzymatic activity, and/or cellular localization can be modulated by the B regulatory subunit.

PP2A is a major serine/threonine protein phosphatase in mammalian cells and has been implicated in the control of numerous biological processes including development, cell growth, differentiation, and apoptosis. It accounts for up to 1% of all cellular proteins and, together with PP1, accounts for 90% of all serine/threonine phosphatase activity in most tissues and cells [18]. It predominantly exists in cells as a heterotrimetic holoenzyme, which consists of a 36 kDa catalytic subunit (PP2A-C), a 65 kDa structural subunit (PP2A-A) forming a core enzyme, and a variable regulatory subunit (PP2A-B), as illustrated in Figure 1[19]. The A structure subunit recruits the C catalytic subunit to form the core dimer, which acts as a scaffold for B subunits of the enzyme. Four B subunit families have been identified (PR55 or B, PR61 or B’, PR72/130 or B” and PR93/PR110 or B”’). Different B subunits interact via the same or overlapping sites within the A subunit of the core dimer. The association of these B subunits with the core AC dimer is mutually exclusive. The PP2A holoenzyme's substrate specificity, enzymatic activity, and/or cellular localization can be modulated by the B regulatory subunit [20-23].

Each family of B subunits contains several isoforms that can bind the AC dimer in a mutually exclusive manner [24]. In mammalian cells, B or PR55 family (α, β, γ and δ) is expressed in a tissue-specific manner. PR55α and PR55δ are widely-distributed in different tissues, whereas PR55β and PR55γ are highly enriched in the brain. PR55α is distributed primarily in the cell body and nucleus of Purkinje cells, whereas PR55β is excluded from the nucleus and extends into dendrites. The B’ family consists of five primary members of the PR61 (α, β, γ, δ and ϵ) that are mapped to the human chromosomes loci 1q41, 11q12, 3p21, 6p21.1, and 7p11.2, respectively. PR61α, PR61β and PR61ϵ localize to the cytoplasm, whereas PR61γ1, PR61γ2, PR61γ3 are concentrated in the nucleus, and PR61δ is found in both the nucleus and the cytoplasma. PR61α and PR61γ1-γ3 are enriched in heart and skeletal muscles. PR61β and PR61δ are expressed predominantly in the brain. For the B” family, PR72 is expressed exclusively in the heart and skeletal muscles, whereas PR130 is expressed predominately in the heart and muscles. The PR93/PR110 comprises the B”’ family. PR110 is localized to the post-synaptic densities of neuronal dendrites, whereas PR93 is nuclear located (Table 1) [16,22]. The spinal cord dorsal horn is an important area containing sensory neurons that has been implicated in major pain processing. Our previous immunofluorescent staining studies have shown that the neurons with PP2A expression are mainly distributed in the superficial layers of the dorsal horn, laminae I and II, and a few of the PP2A immunoreactive neurons are found in the ventral horn of the spinal cord as well (unpublished data).

**Table 1:** Tissue distribution and subcellular localization of PP2A subunits

**Subunits**	**Molecule**	**KDa**	**Isoforms**	**Tissue distribution**	**Subcellular localization**
Structure subunit	A	65	α and β	Ubiquitously expressed	
Catalytic subunit	C	36	α and β	Ubiquitously expressed. High levels in brain and heart.	
Regulatory subunit	B	55	B α	Wide-spread tissue distribution	Cytosolic fraction
			B β	Enriched in brain	Cytosolic fraction
			B δ	Wide-spread tissue distribution	
			B γ	Enriched in brain	Cytoskeletal fraction
			B’ α	Widely expressed and abundant in heart and skeletal muscle	Cytoplasma
			B’ β	Enriched in brain	Cytoplasma
			B’ δ	Enriched in brain	Neucleus and cytoplasma
			B’ γ	Widely expressed and abundant in heart and skeletal muscle	Neucleus
			B’ ϵ		Cytoplasma
			B” PR72	Heart and skeletal muscle	
			B”PR130	Ubiquitously expressed and high levels in heart and muscle	
			B”PR59	Testis, kidney, liver, brain, heart and lung	
			B”PR48		neucleus
			B”’ PR93		neucleus
			B”’PR110		Postsynaptic densities of neuronal dendrites

### The functional regulation of PP2A activity through post-translational modification

Previous studies have demonstrated that modulations of PP2A activity are due to the tyrosine or serine/threonine phosphorylation of not only the catalytic C subunit, but also the regulatory B subunit, especially those of the B’ family [18,20]. The regulatory B subunits play key roles in controlling PP2A substrate specificity, cellular localization, and enzymatic activity [25]. Tyrosine kinases such as Src kinase, inhibit PP2A activity, and the PP2A assembly can be inhibited by the phosphorylation of the B56 subunit by extracellular signaling regulated kinase (ERK) [26,27]. Another post-translational modification of the catalytic C subunit, methylation, which occurs on the carboxy group of the C-terminal residue Leu^309^, is also involved in the alterations of PP2A’s activity [28,29]. It has been shown that the methylation of PP2A-C may influence the affinity of the AC core dimer to the different B subunits [30,31]. For example, some B regulatory subunits appear to bind to an AC dimer more efficiently when the catalytic C subunit has been methylated, whereas other B subunits prefer to bind an AC dimer with a demethylated C subunit [32]. The post-translational modification of PP2A has been implicated in the pathogenesis of Alzheimer’s disease (AD), a neurodegenerative disorder with impaired synaptic plasticity [33-37]. The reduced methylation of PP2A C subunit at Leu^309^ and the increased phosphorylation of PP2A C subunit at Tyr^307^ may result in loss of enzymatic activity and tau hyper-phosphorylation in Alzheimer’s disease, indicating that PP2A is a putative target of therapeutic intervention [34]. How the post-translational modification of PP2A is regulated during nociception is still unclear and deserves further investigations.

### Substrate molecules and signal transduction cascades regulated by PP2A in synaptic plasticity and central sensitization in response to nociceptive stimuli

It has been demonstrated that synaptic glutamate receptors play a critical role in synaptic plasticity, electro-physiologically characterized by long term potentiation (LTP) and long-term depression (LTD), in the central nervous system [38-41]. Previous studies from ours and other groups have demonstrated that central sensitization of pain may represent a spinal form of LTP, since there are close parallels in mechanisms important for LTP and central sensitization [1,38-40] . The functional activation of post-synaptic glutamate receptors may influence a variety of intracellular signals, which may trigger cellular and molecular changes at transcriptional, translational, or post-translational levels. Theses changes contribute to the synaptic plasticity and central sensitization induced by peripheral noxious stimulation [3,6,42,43]. The phosphorylation and de-phosphorylation of synaptic glutamate receptors are important post-translational mechanisms in the modulation of synaptic strength. Strong noxious stimulation in the periphery may activate several protein kinases such as calcium/camodulin dependent protein kinase II (CaMKII), cAMP-dependent protein kinase (PKA), protein kinase C (PKC), and protein kinase G (PKG), which play an important role in the phosphorylation of glutamate receptors in spinal nociceptive neurons [1,3,7,42-45]. The increased sensitivity of glutamate receptors through the phosphorylation regulated by protein kinases may contribute to the enhanced responsiveness of dorsal horn neurons during central sensitization [1,3,10]. In contrast, the protein phosphotase, PP2A may reverse these signals through the de-phosphorylation of glutamate receptors and several intracellular protein kinases, and therefore, blunt the central sensitization of pain [11-14].

### PP2A is involved in the induction and maintenance of synaptic plasticity: electrophysiological evidence

The modification of protein phosphorylation is a critical element leading to the induction of synaptic plasticity. For example, long-term potentiation is accompanied by increased glutamate receptor phosphorylation through various protein kinases and a concomitant decrease in protein phosphatase activity [46]. In contrast, a decrease in synaptic strength, LTD, has been shown to be dependent on glutamate receptor de-phosphorylation mediated by an increase in the activity of protein phosphatases, possibly PP1 and PP2A [47]. Thus, a coordination of kinase and phosphatase activities is crucial for the comprehensive modulation of synaptic plasticity.

**Figure 2:**
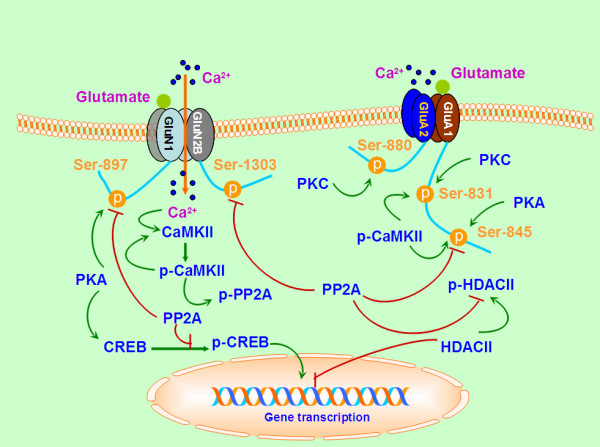
**Molecular substrates regulated by PP2A in synaptic plasticity and central sensitization of pain.** The auto-phosphorylation of CaMKII triggered by the activation of NMDA receptors is an event critical to the induction of LTP. PP2A is a substrate for CaMKII phosphorylation and the phosphorylated PP2A may decrease the activity of PP2A during LTP. The CaMKII-dependent suppression of PP2A activity and prevention of the de-phosphorylation of CaMKII by PP2A may be necessary for the LTP maintenance. PP2A regulates LTP by competing with PKA for the regulation of specific phosphorylation sites, such as the GluN1 subunits of NMDA receptors. AMPA receptor GluA1 subunit has the two major phosphorylation sites: Ser^845^, which is phosporylated by PKA, and Ser ^831^, which is phosphorylated by PKC. CaMKII was also found to phosphorylate both Ser^831^ and Ser^845^ in GluA1, and contributes to the single-channel conductance of the receptor, and thus, possibly increases AMPA receptor conductance during LTP. The de-phosphorylation of Ser^845^ was blocked by the pretreatment with okadaic acid, indicating an involvement of PP1 and/or PP2A. The phosphorylation or de-phosphorylation of AMPA receptors is closely associated with the receptor trafficking. The GluA1 subunit is the important substrate of PP2A, indicating PP2A activity is a critical for AMPA receptor trafficking and might play an important role in AMPA receptor-mediated nociception. The transcription factor cAMP-response-element-binding protein (CREB) has been demonstrated to be involved in synaptic plasticity and gene transcription and PP2A is thought to be the main CREB phosphatase. Histone deacetylase (HDAC) may reverse the action of histone acetylase and block the gene transcription process through the chromatin remodeling. PP2A is responsible for the de-phosphorylation of class II HDACs and for the subsequent triggering nuclear localization and repression of target genes, while the phosphorylaton-triggering cytoplasmatic localization may lead to the activation of target genes.

The application of the PP1/PP2A inhibitor calyculin A, or the post-synaptic injection of microcystin, in hippocampal slices induces a rapid enhancement of synaptic transmission , particularly in the hippocampal tissue from aged rats [48]. It indicates that synaptic transmission can be actively and persistently regulated by protein phosphatases. It has been shown that PKA plays an important role in N-methyl-D-aspartic acid (NMDA) receptor-mediated plasticity in the hippocampus and spinal cord through the phosphorylation of GluN1 subunit of NMDA receptors. Both pharmacological and genetic inhibition of the cAMP/PKA cascade may inhibit the LTP in the hippocampal CA1 area [49,50]. The sensitivity of LTP (elicited by either multiple 100-Hz trains or prolonged 5-Hz stimulation) to PKA inhibition was eliminated by the prior incubation of hippocampal slices with a PP1/PP2A inhibitor, suggesting that the PKA pathway participates in LTP, which may include the activity-dependent suppression of PP1/PP2A activity [51,52]. It has been noted that the induction of LTP is associated with an inhibition of PP2A. The inhibition of PP2A activity was not only observed immediately after the induction of LTP, but was still detectable one hour after induction, indicating that persistently decreased PP2A activity may have a role in the maintenance of LTP [53,54]. Furthermore, the observed inhibition of PP2A showed a pattern of NMDA-receptors dependence. The auto-phosphorylation of CaMKII triggered by the activation of NMDA receptors is a cellular event critical to the induction of LTP. It is shown that the purified PP2A Bα is a substrate for CaMKII phosphorylation, and this subunit is phosphorylated during the induction of LTP in the hippocampus [54]. Combined with these data, we may suggest that the inhibited activity of PP2A during LTP is associated with the phosphorylation of PP2A mediated by CaMKII triggered by the activation of NMDA receptor. As for the decrease in PP2A activity, this phosphorylation persisted for more than one hour after LTP induction. In view of this CaMKII-dependent decrease in PP2A activity, it is interesting that auto-phosphorylated CaMKII is much more readily de-phosphorylated by PP1 than by PP2A [54]. Thus, the CaMKII-dependent suppression of PP2A activity and prevention of the de-phosphorylation of CaMKII by PP2A might serve as key processing events necessary for the LTP maintenance (Figure 2). Pi et al. [55] showed that the coupled PP2A and CaMKII switches lead to a tristable system in which the kinase activity is high in the LTP state, the PP2A activity is high in the LTD state, and neither activity is high in the basal state. These data provide an explanation for the inhibition of PP2A prevents LTD induction.

The auto-phosphorylation of CaMKII induced by tetanization is reported to be blocked by a PKA inhibitor, indicating a potential downstream substrate regulated by the PKA-dependent suppression of phosphatase activity. One possibility is that PP2A regulates LTP by competing with PKA for the regulation of specific phosphorylation sites, such as the GluN1 subunit of NMDA receptors. Although much more work needs to be done in this area, the data indicate an important involvement of the persistent down-regulation of PP2A activity in the maintenance of LTP. PP2A are also found to be involved in the development of LTD in the hippocampus. A number of studies showed that PP2A inhibitors disrupt NMDA receptor-dependent LTD at glutamatergic synapses in hippocampus [56,57].

### PP2A is implicated in synaptic plasticity and pain central sensitization through regulating the function of NMDA receptors

NMDA receptors are nonselective cation channels critical for neuronal excitability and particularly for Ca^2+^-dependent modulation of synaptic plasticity in nociceptive processing. It was found that in the mouse nucleus accumbens, endogenous PP2A regulates NMDA receptor channel phosphorylation, activity and kinetics. In cultured hippocampal neurons, exogenous PP2A depressed the open probability of NMDA receptors [58]. Phosphatase may participate in the long-term changes in NMDA receptor function, such as LTP or LTD. Okadaic acid, which serves as a potent and specific inhibitor of the serine/threonine protein phosphatases 1 and 2A, may enhance the open probability of NMDA receptors [59,60]. It has been demonstrated that okadaic acid increases the NMDA and AMPA-kainate receptors-mediated currents in neurons of the hippocampus [61]. Intra-hippocampal micro-injection of okadaic acid induces the hyper-phosphorylation of the GluN2B subunit of the NMDA receptors [62]. Association of PP2A with NMDA receptors may result in an increase in the phosphatase activity of PP2A and the dephosphorylation of Serine^897^ of the NMDA receptor subunit NR1. On the other hand, the dissociation of PP2A from the complex and the reduction of PP2A activity can be caused by stimulation of NMDA receptors [63].

Using an animal model of inflammatory pain, our previous studies have demonstrated that PP2A plays a critical role in determining the excitability of nociceptive neurons in the spinal cord by modulating the phosphorylation state of some critical proteins [13,14]. Infusion of a general inhibitor of PP2A, okadaic acid (OA), and a specific inhibitor, fostriecin into the subarachnoid space may significantly enhance the secondary mechanical hyperalgesia and allodynia following intradermal injection of capsaicin [14]. Further, we found that the up-regulated phosphorylation of both GluN1 and GluN2B subunits of NMDA receptors induced by capsaicin injection was significantly potentiated by the PP2A inhibitor without affecting the GluN1 and GluN2B protein itself in the spinal cord dorsal horns. It suggests that PP2A may have a regulatory effect on central sensitization induced by noxious stimuli in the periphery by regulating the phosphorylation state of NMDA receptors [13]. Using an *in vivo* electrophysiological recording technique, our previous study has also shown that PP2A inhibitors significantly prolonged the responses of dorsal horn neurons to mechanical stimuli in anesthetized rats following intradermal injection of capsaicin, indicating PP2A may be involved in determining the duration of capsaicin-induced central sensitization [11,12].

### PP2A regulates the function of AMPA receptor through influencing the dephosphorylation and trafficking of AMPA receptor subunits

AMPA receptor has an important role in synaptic plasticity and central sensitization of pain [8,9,64-66]. AMPA receptors present unique functional regulations, such as subunits phosphorylation and membrane insertion or internalization. The intracellular C-terminal domains of AMPA receptor subunits allow subunit-specific regulation by phosphorylation. There are several protein phosphorylation sites located on the C-terminal region, which are working targets of PKA, PKC, and CaMKII [1,7]. Site-directed mutagenesis and phosphopeptide analysis identified two major phosphorylation sites on GluR1: Ser^845^, which is phosporylated by PKA, and Ser ^831^, which is phosphorylated by PKC. The phosphorylation of Ser^845^ in GluA1 by PKA regulates the open-channel probability of AMPA receptors; whereas the phosphorylation of Ser^831^ by PKC changes channel conductance. CaMKII was also found to phosphorylate both Ser^831^ and Ser^845^ in GluA1 and contributes to the single-channel conductance of the receptor and possibly increases AMPA receptor conductance during LTP [65]. The phosphorylation of GluA2 plays an important role in the receptor clusters during synaptic plasticity and persistent painful stimulation [67]. It has been demonstrated that GluA2 may be phosphorylated on Ser^880^ by PKC *in vitro* and in transfected cells [68].

The development of phosphorylation-site-specific antibodies has greatly facilitated the study of the phosphorylation state of endogenous proteins regulated by kinases and phosphatases. Huganir’s group has shown that the induction of LTD by prolonged, low-frequency stimulation led to a decrease in the phosphorylation of Ser^845^ but not Ser^831^ of GluA1 subunits, as assessed by the Western blot analysis of hippocampal slices after stimulation [69]. The de-phosphorylation of Ser^845^ was blocked by pre-treatment with okadaic acid, indicating an involvement of PP1 and/or PP2A (Figure 2). So, GluA1 is a critical substrate of protein phosphatases in LTD. Although a similar decrease in Ser^845^ phosphorylation was observed when LTD was induced chemically by the application of NMDA, this de-phosphorylation was not blocked by high concentrations of either okadaic acid or calyculin A [70]. This result indicates that different populations of GluA1 might be regulated by distinct phosphatases. Consistent with this idea, Strack and colleagues have observed a similar phenomenon in the case of another substrate, CaMKII. Whereas soluble CaMKIIα is a PP2A substrate, translocation of CaMKIIα to the postsynaptic density by auto-phosphorylation converts it to a PP1 substrate [54]. Interestingly, it is noted that CaMKII activity in the postsynaptic density does not appear to correlate with synaptic insertion of GluA1 at C fiber synapses in inflammatory pain [41,71]. Thus, this strongly supports the notion that, even if PP1 is the major phosphatase in the postsynaptic density, cytosolic PP2A (or PP2B) may regulate the auto-phosphorylation of CaMKII that mediates synaptic AMPA receptor incorporation. However, the synaptic induction of LTD is associated with the de-phosphorylation of Ser^845^, but not Ser^831^, and the induction of LTP in naive slices is observed to be associated with an increase in the phosphorylation of Ser^831^, but not Ser^845^. LTP-inducing stimulation only elicited increased Ser^845^ phosphorylation when LTP was induced at previously depressed synapses. Conversely, at previously potentiated synapses, the administration of de-potentiating, low-frequency stimuli produced de-phosphorylation of Ser^831^, but not Ser^845^[69,72,73]. These studies show that the de-phosphorylation regulation by protein phosphotases in synaptic plasticity is associated with the functional status of the synapses. In spinal neurons, our group has shown that PKA mediates the phosphorylation of serine at the Ser^845^ site, and PKC targets the Ser^831^ site following noxious stimulation [7]. Further, we have demonstrated that AMPA receptors showed enhanced responsiveness to nociceptive stimulation through this phosphorylation step during central sensitization [1]. However, much less is known about the de-phosphorylation regulation of GluA1 or GluA2 subunits by PP2A in different pain models.

The de-phosphorylation of ligand-gated channels regulates the channel properties. The accumulated evidence indicates that phosphatase activity also regulates the surface insertion or cluster of these neuronal receptors. Several studies have shown that NMDA-receptor-dependent LTD is associated with a post-synaptic silence of synapses, and that a post-synaptic interference with the endocytotic machinery hampers LTD. A series of studies now show that the rapid trafficking of AMPA receptors may occur in the hippocampal or spinal dorsal horn neurons after subunit phosphorylaiton [74,75]. The internalization of AMPA receptors can be blocked by inhibitors of protein phosphotase, indicating that protein phosphatase might have a regulatory role in LTD [76]. Currently, little is known about the role of PP2A in the trafficking of AMPA receptor subunits. It has been demonstrated that a membrane insertion of Ca^2+^-permeable AMPA receptors greatly contributes to the synaptic plasticity in hippocampus and central sensitization of pain in the spinal cord dorsal horns [8,77]. Huganir’s group has also shown that the trafficking of AMPA receptors is regulated through the PKA phosphorylation of GluA1 subunits [78,79]. Another study indicated that the signaling pathway that drive the insertion of GluA1 subunits into the plasma membrane during LTP *in vitro* require the activated CaMKII [80]. In spinal neurons, intrathecal application of a CaMKII inhibitor, KN-93, before the painful visceral stimulus, apparently inhibits the GluA1 accumulation in the plasma membrane fraction. Peripheral inflammation stimuli drive the phosphorylation and trafficking of AMPA receptor subunits in spinal cord dorsal horns [81,82]. The data suggests that the phosphorylation or de-phosphorylation of AMPA receptors is closely associated with the receptor trafficking events. The GluA1 subunit is an important substrate of PP2A, indicating activated PP2A is a critical modulator of AMPA receptor trafficking and might play an important role in AMPA receptor-mediated nociception (Figure 2). In future studies, it will be valuable to determine the activity status of PP2A in the spinal cord dorsal horns and the de-sphosphorylation as well as trafficking regulation of AMPA receptor GluA1 or GluA2 subunits by PP2A in different animal models of pain.

### PP2A regulates synaptic transmission through influencing transcription factors and subsequent chromatin remodeling

Another way in which phosphatases might regulate synaptic transmission on a longer timescale is through the alternation of chromatin remodeling and gene transcription. Calcium-dependent gene transcription has a critical role in both synaptic plasticity and memory formation. The transcription factor cAMP-response-element-binding protein (CREB) has been demonstrated to be involved in synaptic plasticity and long-term memory, and serves a substrate for various phosphatases as well [57]. In cultured hippocampal neurons, PP1 and/or PP2A are observed to be main CREB phosphatases, and although PP2B does not directly de-phosphorylate CREB, it nonetheless has a key role in regulating CREB-mediated transcription process [83]. Various stimuli evoke the transient phosphorylation of CREB protein, but a more sustained phosphorylation seems to be necessary for a CREB-mediated transcription to occur. It has been shown that PP2A may play a critical role in constraining the progression of information from the synapse to the nucleus (Figure 2). It will be important to learn how the activity dependent regulation of PP2A influences the relevant gene expression. PP2A has been shown to form a signaling complex with CaMKIV that regulates CREB phosphorylation, and thus, a CREB-mediated transcription [51]. PP2A holoenzymes may also negatively regulate NF-κB-mediated transcriptional activities [84]. Some PP2A regulatory subunits, such as PR55γ and PR55δ, are inhibitors of JNK and c-Src kinases, which are important to regulate transcription factors.

The important role for multiple protein kinases in regulation of nociception in animal studies suggests its function on nociception-elicited gene expression through mediation of transcription factors, such as *c-fos* and CREB. Increased phosphorylation of CREB protein through the activation of glutamate receptors and the PKA, PKC, and CaMKII cascades during central sensitization was reported in several animal models of pain [2,6,10,42]. It suggests an intra-cellular connection between activation of transcription factors and molecular mechanisms mediating stimuli-induced nuclear gene activation through several protein kinase pathways. PP2A may exert a negative action on CREB-mediated transcription-dependent central sensitization, since CREB is an important substrate of PP2A. Future investigations need to determine the regulation of PP2A activity during central sensitization through multiple transcription factors, such as *c-fos*, *c-Jun* and NF-κB.

Previous studies have suggested that post-translational or epi-genetic modification (acetylation, methylation, phosphorylation, etc.) of histones, and subsequently remodeling of chromatin structure, play a critical role in controlling gene transcription and facilitating long-term changes during synaptic plasticity and central sensitization of pain [85-88]. Chromatin structures are regulated in hippocampal and spinal neurons in response to activation of multiple kinase activation. In particular, it has been shown that the histone acetyltransferase activity of CREB binding protein (CBP) is necessary for synaptic plasticity and central sensitization of pain [89]. CREB can be phosphorylated and activated by different kinases and then it recruits the histone acetyltransferase co-activator CBP and its homologue p300. The recruitment of CBP/p300 and changes in the level of histone acetylation are required for gene transcription. Another study from our group reported that the activated JNK signaling pathway was observed to contribute to the regulation of histone remodeling in peripheral sensory neurons following neurotoxic stimulation [90]. Therefore, PP2A activity may regulate the histone-acetylation induced subsequent neuro-epigenetic changes and downstream gene transcription through the de-phosphorylation of CREB.

In contrast to histone acetylase, histone deacetylase (HDAC) may remove acetyl groups from lysine residues of histones, and other non-histone proteins, and reverse the action of histone acetylase and block the gene transcription process. PP2A comprises a family of holoenzyme complexes with diverse biological activities, which mainly depend on individual regulatory subunits. The PP2A heterotrimeric complex was formed by the PP2A-A subunit and the catalytic subunit (PP2A-C), while G5PR as a regulatory subunit exhibits phosphatase activity on histone H1. Class II HDACs are key transcriptional regulators whose activities are controlled via phosphorylation-dependent nucleo-cytoplasmic shuttling [91]. PP2A is responsible for de-phosphorylation of class II HDACs triggering nuclear localization and repression of target genes, whereas phosphorylation triggers cytoplasmatic localization leading to activation of target genes [91] (Figure 2). Recent studies have shown that the HDACs have been implicated in spinal nociception in inflammatory pain [92,93]. It is presumable that PP2A may regulate the gene expression in the spinal cord dorsal horns elicited by peripheral noxious stimuli through the de-phosphorylation of class II HDACs and subsequent neuro-epigenetic alternations.

## Concluding remark

In this review, we have highlighted the roles of PP2A in synaptic plasticity and central sensitization of nociceptive process. Specifically, they have key roles in limiting LTP induction and maintenance, and in triggering LTD induction. PP2A activity is regulated by holoenzyme composition, post-translational modification of methylation and phosphorylation. PP2A may exert its action through the de-phosphorylaiton of critical substrates relevant to nociceptive processing, such as NMDA receptor subunits, AMPA receptor GluA1 subunits, transcription factors and class II HDACs. All these substrate molecules are implicated in synaptic plasticity and central sensitization in the central nervous system. PP2A may serve as a potential molecular target that can be selectively and effectively modulated through pharmaceutical intervention to treat pain.

## Competing interests

The authors declare that they have no competing interests.

## Authors’ contributions

YW participated in the design of the review and drafted the manuscript. YL, JW and YM assisted with the preparation of the manuscript and figures. LF, and XZ conceived of the review and participated in its design and helped to draft the manuscript. All authors read and approved the final manuscript.

## References

[B1] Fang L Wu J Lin Q Willis WD Calcium-calmodulin-dependent protein kinase II contributes to spinal cord central sensitization J Neurosci 2002 22 4196 4204 12019337 10.1523/JNEUROSCI.22-10-04196.2002 PMC6757653

[B2] Wu J Su G Ma L Zhang X Lei Y Li J Lin Q Fang L Protein kinases mediate increment of the phosphorylation of cyclic AMP-responsive element binding protein in spinal cord of rats following capsaicin injection Mol Pain 2005 1 26 10.1186/1744-8069-1-26 16159392 PMC1224868

[B3] Wu J Li J Lin Q Fang L Signal transduction in chronic pain Int Anesthesiol Clin 2007 45 73 81 10.1097/AIA.0b013e31803419f7 17426509

[B4] Faure C Ramos M Girault JA Pyk2 cytonuclear localization: mechanisms and regulation by serine dephosphorylation Cell Mol Life Sci 2013 70 137 152 10.1007/s00018-012-1075-5 22802128 PMC11113809

[B5] Mizuno K Signaling mechanisms and functional roles of cofilin phosphorylation and dephosphorylation Cell Signal 2013 25 457 469 10.1016/j.cellsig.2012.11.001 23153585

[B6] Fang L Wu J Zhang X Lin Q Willis WD Calcium/calmodulin dependent protein kinase II regulates the phosphorylation of cyclic AMP-responsive element-binding protein of spinal cord in rats following noxious stimulation Neurosci Lett 2005 374 1 4 10.1016/j.neulet.2004.10.014 15631885

[B7] Fang L Wu J Lin Q Willis WD Protein kinases regulate the phosphorylation of the GluR1 subunit of AMPA receptors of spinal cord in rats following noxious stimulation Brain Res Mol Brain Res 2003 118 160 165 10.1016/j.molbrainres.2003.08.002 14559367

[B8] Wang Y Wu J Wu Z Lin Q Yue Y Fang L Regulation of AMPA receptors in spinal nociception Mol Pain 2010 6 5 10.1186/1744-8069-6-5 20092646 PMC2823608

[B9] Wang Y Mu X Wu J Wu A Fang L Li J Yue Y Differential roles of phosphorylated AMPA receptor GluR1 subunits at Serine-831 and Serine-845 sites in spinal cord dorsal horn in a rat model of post-operative pain Neurochem Res 2011 36 170 176 10.1007/s11064-010-0288-y 20953906

[B10] Wu J Fang L Lin Q Willis WD The role of nitric oxide in the phosphorylation of cyclic adenosine monophosphate-responsive element-binding protein in the spinal cord after intradermal injection of capsaicin J. Pain 2002 3 190 198 10.1054/jpai.2002.123653 14622772

[B11] Zhang X Wu J Fang L Willis WD The effects of protein phosphatase inhibitors on the duration of central sensitization of rat dorsal horn neurons following injection of capsaicin Mol Pain 2006 2 23 10.1186/1744-8069-2-23 16846502 PMC1559591

[B12] Zhang X Wu J Lei Y Fang L Willis WD Protein phosphatase 2A regulates central sensitization in the spinal cord of rats following intradermal injection of capsaicin Mol Pain 2006 2 9 10.1186/1744-8069-2-9 16549018 PMC1456949

[B13] Zhang X Wu J Lei Y Fang L Willis WD Protein phosphatase modulates the phosphorylation of spinal cord NMDA receptors in rats following intradermal injection of capsaicin Brain Res Mol Brain Res 2005 138 264 272 10.1016/j.molbrainres.2005.05.001 15919130

[B14] Zhang X Wu J Fang L Willis WD The effects of protein phosphatase inhibitors on nociceptive behavioral responses of rats following intradermal injection of capsaicin Pain 2003 106 443 451 10.1016/j.pain.2003.09.002 14659528

[B15] Gabra BH Bailey CP Kelly E Sanders AV Henderson G Smith FL Dewey WL Evidence for an important role of protein phosphatases in the mechanism of morphine tolerance Brain Res 2007 1159 86 93 17582387 10.1016/j.brainres.2007.05.017 PMC3736353

[B16] Zhang Q Claret FX Phosphatases: the new brakes for cancer development? Enzyme Res 2012 2012 659649 22121480 10.1155/2012/659649 PMC3206369

[B17] Winder DG Sweatt JD Roles of serine/threonine phosphatases in hippocampal synaptic plasticity Nat Rev Neurosci 2001 2 461 474 10.1038/35081514 11433371

[B18] Janssens V Goris J Protein phosphatase 2A: a highly regulated family of serine/threonine phosphatases implicated in cell growth and signalling Biochem J 2001 353 417 439 10.1042/0264-6021:3530417 11171037 PMC1221586

[B19] Janssens V Longin S Goris J PP2A holoenzyme assembly: in cauda venenum (the sting is in the tail) Trends Biochem Sci 2008 33 113 121 10.1016/j.tibs.2007.12.004 18291659

[B20] Janssens V Derua R Zwaenepoel K Waelkens E Goris J Specific regulation of protein phosphatase 2A PR72/B'' subunits by calpain Biochem Biophys Res Commun 2009 386 676 681 10.1016/j.bbrc.2009.06.096 19555667

[B21] Park JH Sung HY Lee JY Kim HJ Ahn JH Jo I B56alpha subunit of protein phosphatase 2A mediates retinoic acid-induced decreases in phosphorylation of endothelial nitric oxide synthase at serine 1179 and nitric oxide production in bovine aortic endothelial cells Biochem Biophys Res Commun 2013 430 476 481 10.1016/j.bbrc.2012.12.011 23237802

[B22] Zwaenepoel K Louis JV Goris J Janssens V Diversity in genomic organisation, developmental regulation and distribution of the murine PR72/B" subunits of protein phosphatase 2A BMC. Genomics 2008 9 393 10.1186/1471-2164-9-393 18715506 PMC2529318

[B23] Ahn JH Sung JY McAvoy T Nishi A Janssens V Goris J Greengard P Nairn AC The B''/PR72 subunit mediates Ca2+−dependent dephosphorylation of DARPP-32 by protein phosphatase 2A Proc Natl Acad Sci U S A 2007 104 9876 9881 10.1073/pnas.0703589104 17535922 PMC1887582

[B24] Lechward K Awotunde OS Swiatek W Muszynska G Protein phosphatase 2A: variety of forms and diversity of functions Acta Biochim Pol 2001 48 921 933 11996003

[B25] Cho US Morrone S Sablina AA Arroyo JD Hahn WC Xu W Structural basis of PP2A inhibition by small t antigen PLoS Biol 2007 5 e202 10.1371/journal.pbio.0050202 17608567 PMC1945078

[B26] Letourneux C Rocher G Porteu F B56-containing PP2A dephosphorylate ERK and their activity is controlled by the early gene IEX-1 and ERK EMBO J 2006 25 727 738 10.1038/sj.emboj.7600980 16456541 PMC1383561

[B27] Chen J Martin BL Brautigan DL Regulation of protein serine-threonine phosphatase type-2A by tyrosine phosphorylation Science 1992 257 1261 1264 10.1126/science.1325671 1325671

[B28] Longin S Zwaenepoel K Martens E Louis JV Rondelez E Goris J Janssens V Spatial control of protein phosphatase 2A (de)methylation Exp Cell Res 2008 314 68 81 10.1016/j.yexcr.2007.07.030 17803990

[B29] Longin S Zwaenepoel K Louis JV Dilworth S Goris J Janssens V Selection of protein phosphatase 2A regulatory subunits is mediated by the C terminus of the catalytic Subunit J Biol Chem 2007 282 26971 26980 10.1074/jbc.M704059200 17635907

[B30] Ogris E Gibson DM Pallas DC Protein phosphatase 2A subunit assembly: the catalytic subunit carboxy terminus is important for binding cellular B subunit but not polyomavirus middle tumor antigen Oncogene 1997 15 911 917 10.1038/sj.onc.1201259 9285686

[B31] Bryant JC Westphal RS Wadzinski BE Methylated C-terminal leucine residue of PP2A catalytic subunit is important for binding of regulatory Balpha subunit Biochem J 1999 339 Pt 2 241 246 10191253 PMC1220151

[B32] Leulliot N Quevillon-Cheruel S Sorel I de La Sierra-Gallay L Collinet B Graille M Blondeau K Bettache N Poupon A Janin J van TH Structure of protein phosphatase methyltransferase 1 (PPM1), a leucine carboxyl methyltransferase involved in the regulation of protein phosphatase 2A activity J Biol Chem 2004 279 8351 8358 14660564 10.1074/jbc.M311484200

[B33] Rudrabhatla P Pant HC Role of protein phosphatase 2A in Alzheimer's disease Curr Alzheimer Res 2011 8 623 632 10.2174/156720511796717168 21605044

[B34] Torrent L Ferrer I PP2A and Alzheimer disease Curr Alzheimer Res 2012 9 248 256 10.2174/156720512799361682 22299660

[B35] Sun XY Wei YP Xiong Y Wang XC Xie AJ Wang XL Yang Y Wang Q Lu YM Liu R Wang JZ Synaptic released zinc promotes tau hyperphosphorylation by inhibition of protein phosphatase 2A (PP2A) J Biol Chem 2012 287 11174 11182 10.1074/jbc.M111.309070 22334661 PMC3322889

[B36] Sontag E Hladik C Montgomery L Luangpirom A Mudrak I Ogris E White CL III Downregulation of protein phosphatase 2A carboxyl methylation and methyltransferase may contribute to Alzheimer disease pathogenesis J Neuropathol Exp Neurol 2004 63 1080 1091 15535135 10.1093/jnen/63.10.1080

[B37] Sontag E Luangpirom A Hladik C Mudrak I Ogris E Speciale S White CL III Altered expression levels of the protein phosphatase 2A ABalphaC enzyme are associated with Alzheimer disease pathology J Neuropathol Exp Neurol 2004 63 287 301 15099019 10.1093/jnen/63.4.287

[B38] Sandkuhler J Gruber-Schoffnegger D Hyperalgesia by synaptic long-term potentiation (LTP): an update Curr Opin Pharmacol 2012 12 18 27 10.1016/j.coph.2011.10.018 22078436 PMC3315008

[B39] Sandkuhler J Central sensitization versus synaptic long-term potentiation (LTP): a critical comment J Pain 2010 11 798 800 10.1016/j.jpain.2010.05.002 20674850

[B40] Larsson M Broman J Synaptic plasticity and pain: role of ionotropic glutamate receptors Neuroscientist 2011 17 256 273 10.1177/1073858409349913 20360599

[B41] Larsson M Broman J Translocation of GluR1-containing AMPA receptors to a spinal nociceptive synapse during acute noxious stimulation J Neurosci 2008 28 7084 7090 10.1523/JNEUROSCI.5749-07.2008 18614677 PMC6670484

[B42] Wu J Su G Ma L Zhang X Lei Y Lin Q Nauta HJ Li J Fang L The role of c-AMP-dependent protein kinase in spinal cord and post synaptic dorsal column neurons in a rat model of visceral pain Neurochem Int 2007 50 710 718 10.1016/j.neuint.2007.01.006 17320244 PMC1894916

[B43] Willis WD Role of neurotransmitters in sensitization of pain responses Ann N Y Acad Sci 2001 933 142 156 12000017 10.1111/j.1749-6632.2001.tb05821.x

[B44] Tanabe M Nagatani Y Saitoh K Takasu K Ono H Pharmacological assessments of nitric oxide synthase isoforms and downstream diversity of NO signaling in the maintenance of thermal and mechanical hypersensitivity after peripheral nerve injury in mice Neuropharmacology 2009 56 702 708 10.1016/j.neuropharm.2008.12.003 19111753

[B45] Schmidtko A Ruth P Geisslinger G Tegeder I Inhibition of cyclic guanosine 5'-monophosphate-dependent protein kinase I (PKG-I) in lumbar spinal cord reduces formalin-induced hyperalgesia and PKG upregulation Nitric 2003 8 89 94 10.1016/S1089-8603(02)00165-9 12620371

[B46] Bliss TV Collingridge GL A synaptic model of memory: long-term potentiation in the hippocampus Nature 1993 361 31 39 10.1038/361031a0 8421494

[B47] Thiels E Norman ED Barrionuevo G Klann E Transient and persistent increases in protein phosphatase activity during long-term depression in the adult hippocampus in vivo Neuroscience 1998 86 1023 1029 10.1016/S0306-4522(98)00135-3 9697109

[B48] Figurov A Boddeke H Muller D Enhancement of AMPA-mediated synaptic transmission by the protein phosphatase inhibitor calyculin A in rat hippocampal slices Eur J Neurosci 1993 5 1035 1041 10.1111/j.1460-9568.1993.tb00956.x 7506616

[B49] Blitzer RD Wong T Nouranifar R Iyengar R Landau EM Postsynaptic cAMP pathway gates early LTP in hippocampal CA1 region Neuron 1995 15 1403 1414 10.1016/0896-6273(95)90018-7 8845163

[B50] Winder DG Mansuy IM Osman M Moallem TM Kandel ER Genetic and pharmacological evidence for a novel, intermediate phase of long-term potentiation suppressed by calcineurin Cell 1998 92 25 37 10.1016/S0092-8674(00)80896-X 9489697

[B51] Blitzer RD Connor JH Brown GP Wong T Shenolikar S Iyengar R Landau EM Gating of CaMKII by cAMP-regulated protein phosphatase activity during LTP Science 1998 280 1940 1942 10.1126/science.280.5371.1940 9632393

[B52] Makhinson M Chotiner JK Watson JB O'Dell TJ Adenylyl cyclase activation modulates activity-dependent changes in synaptic strength and Ca2+/calmodulin-dependent kinase II autophosphorylation J Neurosci 1999 19 2500 2510 10087064 10.1523/JNEUROSCI.19-07-02500.1999 PMC6786061

[B53] Fukunaga K Muller D Ohmitsu M Bako E DePaoli-Roach AA Miyamoto E Decreased protein phosphatase 2A activity in hippocampal long-term potentiation J Neurochem 2000 74 807 817 10646534 10.1046/j.1471-4159.2000.740807.x

[B54] Strack S Choi S Lovinger DM Colbran RJ Translocation of autophosphorylated calcium/calmodulin-dependent protein kinase II to the postsynaptic density J Biol Chem 1997 272 13467 13470 10.1074/jbc.272.21.13467 9153188

[B55] Pi HJ Lisman JE Coupled phosphatase and kinase switches produce the tristability required for long-term potentiation and long-term depression J Neurosci 2008 28 13132 13138 10.1523/JNEUROSCI.2348-08.2008 19052204 PMC2620235

[B56] Mulkey RM Herron CE Malenka RC An essential role for protein phosphatases in hippocampal long-term depression Science 1993 261 1051 1055 10.1126/science.8394601 8394601

[B57] Mauna JC Miyamae T Pulli B Thiels E Protein phosphatases 1 and 2A are both required for long-term depression and associated dephosphorylation of cAMP response element binding protein in hippocampal area CA1 in vivo Hippocampus 2011 21 1093 1104 10.1002/hipo.20823 20824729 PMC3046325

[B58] Woo NH Nguyen PV "Silent" metaplasticity of the late phase of long-term potentiation requires protein phosphatases Learn Mem 2002 9 202 213 10.1101/lm.498402 12177233 PMC182582

[B59] Jouvenceau A Billard JM Haditsch U Mansuy IM Dutar P Different phosphatase-dependent mechanisms mediate long-term depression and depotentiation of long-term potentiation in mouse hippocampal CA1 area Eur J Neurosci 2003 18 1279 1285 10.1046/j.1460-9568.2003.02831.x 12956726

[B60] Tapia R Pena F Arias C Neurotoxic and synaptic effects of okadaic acid, an inhibitor of protein phosphatases Neurochem Res 1999 24 1423 1430 10.1023/A:1022588808260 10555783

[B61] Huang CC Liang YC Hsu KS Characterization of the mechanism underlying the reversal of long term potentiation by low frequency stimulation at hippocampal CA1 synapses J Biol Chem 2001 276 48108 48117 11679581 10.1074/jbc.M106388200

[B62] Arias C Montiel T Pena F Ferrera P Tapia R Okadaic acid induces epileptic seizures and hyperphosphorylation of the NR2B subunit of the NMDA receptor in rat hippocampus in vivo Exp Neurol 2002 177 284 291 10.1006/exnr.2002.7988 12429230

[B63] Chan SF Sucher NJ An NMDA receptor signaling complex with protein phosphatase 2A J Neurosci 2001 21 7985 7992 11588171 10.1523/JNEUROSCI.21-20-07985.2001 PMC6763850

[B64] Atianjoh FE Yaster M Zhao X Takamiya K Xia J Gauda EB Huganir RL Tao YX Spinal cord protein interacting with C kinase 1 is required for the maintenance of complete Freund's adjuvant-induced inflammatory pain but not for incision-induced post-operative pain Pain 2010 151 226 234 10.1016/j.pain.2010.07.017 20696523 PMC2939307

[B65] Kristensen AS Jenkins MA Banke TG Schousboe A Makino Y Johnson RC Huganir R Traynelis SF Mechanism of Ca2+/calmodulin-dependent kinase II regulation of AMPA receptor gating Nat Neurosci 2011 14 727 735 10.1038/nn.2804 21516102 PMC3102786

[B66] Anggono V Huganir RL Regulation of AMPA receptor trafficking and synaptic plasticity Curr Opin Neurobiol 2012 22 461 469 10.1016/j.conb.2011.12.006 22217700 PMC3392447

[B67] Park JS Voitenko N Petralia RS Guan X Xu JT Steinberg JP Takamiya K Sotnik A Kopach O Huganir RL Tao YX Persistent inflammation induces GluR2 internalization via NMDA receptor-triggered PKC activation in dorsal horn neurons J Neurosci 2009 29 3206 3219 10.1523/JNEUROSCI.4514-08.2009 19279258 PMC2664544

[B68] Clem RL Anggono V Huganir RL PICK1 regulates incorporation of calcium-permeable AMPA receptors during cortical synaptic strengthening J Neurosci 2010 30 6360 6366 10.1523/JNEUROSCI.6276-09.2010 20445062 PMC2897179

[B69] Lee HK Kameyama K Huganir RL Bear MF NMDA induces long-term synaptic depression and dephosphorylation of the GluR1 subunit of AMPA receptors in hippocampus Neuron 1998 21 1151 1162 10.1016/S0896-6273(00)80632-7 9856470

[B70] Kameyama K Lee HK Bear MF Huganir RL Involvement of a postsynaptic protein kinase A substrate in the expression of homosynaptic long-term depression Neuron 1998 21 1163 1175 10.1016/S0896-6273(00)80633-9 9856471

[B71] Larsson M Broman J Pathway-specific bidirectional regulation of Ca2+/calmodulin-dependent protein kinase II at spinal nociceptive synapses after acute noxious stimulation J Neurosci 2006 26 4198 4205 10.1523/JNEUROSCI.0352-06.2006 16624940 PMC6674005

[B72] Lee HK Barbarosie M Kameyama K Bear MF Huganir RL Regulation of distinct AMPA receptor phosphorylation sites during bidirectional synaptic plasticity Nature 2000 405 955 959 10.1038/35016089 10879537

[B73] Lee HK Takamiya K Han JS Man H Kim CH Rumbaugh G Yu S Ding L He C Petralia RS Wenthold RJ Gallagher M Huganir RL Phosphorylation of the AMPA receptor GluR1 subunit is required for synaptic plasticity and retention of spatial memory Cell 2003 112 631 643 10.1016/S0092-8674(03)00122-3 12628184

[B74] Lu W Roche KW Posttranslational regulation of AMPA receptor trafficking and function Curr Opin Neurobiol 2012 22 470 479 10.1016/j.conb.2011.09.008 22000952 PMC3279598

[B75] Peng HY Chen GD Hsieh MC Lai CY Huang YP Lin TB Spinal SGK1/GRASP-1/Rab4 is involved in complete Freund's adjuvant-induced inflammatory pain via regulating dorsal horn GluR1-containing AMPA receptor trafficking in rats Pain 2012 153 2380 2392 10.1016/j.pain.2012.08.004 22980744

[B76] Beattie EC Carroll RC Yu X Morishita W Yasuda H von ZM Malenka RC Regulation of AMPA receptor endocytosis by a signaling mechanism shared with LTD Nat Neurosci 2000 3 1291 1300 10.1038/81823 11100150

[B77] Clem RL Huganir RL Calcium-permeable AMPA receptor dynamics mediate fear memory erasure Science 2010 330 1108 1112 10.1126/science.1195298 21030604 PMC3001394

[B78] Esteban JA Shi SH Wilson C Nuriya M Huganir RL Malinow R PKA phosphorylation of AMPA receptor subunits controls synaptic trafficking underlying plasticity Nat Neurosci 2003 6 136 143 10.1038/nn997 12536214

[B79] Man HY Sekine-Aizawa Y Huganir RL Regulation of {alpha}-amino-3-hydroxy-5-methyl-4-isoxazolepropionic acid receptor trafficking through PKA phosphorylation of the Glu receptor 1 subunit Proc Natl Acad Sci USA 2007 104 3579 3584 10.1073/pnas.0611698104 17360685 PMC1805611

[B80] Hayashi Y Shi SH Esteban JA Piccini A Poncer JC Malinow R Driving AMPA receptors into synapses by LTP and CaMKII: requirement for GluR1 and PDZ domain interaction Science 2000 287 2262 2267 10.1126/science.287.5461.2262 10731148

[B81] Tao YX AMPA receptor trafficking in inflammation-induced dorsal horn central sensitization Neurosci Bull 2012 28 111 120 10.1007/s12264-012-1204-z 22466122 PMC3324122

[B82] Choi JI Svensson CI Koehrn FJ Bhuskute A Sorkin LS Peripheral inflammation induces tumor necrosis factor dependent AMPA receptor trafficking and Akt phosphorylation in spinal cord in addition to pain behavior Pain 2010 149 243 253 10.1016/j.pain.2010.02.008 20202754 PMC2860679

[B83] Huang L Balsara RD Sheng Z Castellino FJ Conantokins inhibit NMDAR-dependent calcium influx in developing rat hippocampal neurons in primary culture with resulting effects on CREB phosphorylation Mol Cell Neurosci 2010 45 163 172 10.1016/j.mcn.2010.06.007 20600930 PMC2923249

[B84] Hsieh CY Hsu MJ Hsiao G Wang YH Huang CW Chen SW Jayakumar T Chiu PT Chiu YH Sheu JR Andrographolide enhances nuclear factor-kappaB subunit p65 Ser536 dephosphorylation through activation of protein phosphatase 2A in vascular smooth muscle cells J Biol Chem 2011 286 5942 5955 10.1074/jbc.M110.123968 21169355 PMC3057811

[B85] Denk F McMahon SB Chronic pain: emerging evidence for the involvement of epigenetics Neuron 2012 73 435 444 10.1016/j.neuron.2012.01.012 22325197 PMC3996727

[B86] Sultan FA Day JJ Epigenetic mechanisms in memory and synaptic function Epigenomics 2011 3 157 181 10.2217/epi.11.6 22122279 PMC3350307

[B87] Geranton SM Targeting epigenetic mechanisms for pain relief Curr Opin Pharmacol 2012 12 35 41 10.1016/j.coph.2011.10.012 22056026

[B88] Wang Y Chen Z Zhao Y Shi R Wang Y Xu J Wu A Johns RA Yue Y Epigenetics as a new therapeutic target for postoperative cognitive dysfunction Med Hypotheses 2013 80 249 251 10.1016/j.mehy.2012.11.041 23265361

[B89] Zhu XY Huang CS Li Q Chang RM Song ZB Zou WY Guo QL p300 exerts an epigenetic role in chronic neuropathic pain through its acetyltransferase activity in rats following chronic constriction injury (CCI) Mol. Pain 2012 8 84 10.1186/1744-8069-8-84 23176208 PMC3558366

[B90] Wu J Zhang X Nauta HJ Lin Q Li J Fang L JNK1 regulates histone acetylation in trigeminal neurons following chemical stimulation Biochem Biophys Res Commun 2008 376 781 786 10.1016/j.bbrc.2008.09.073 18822271 PMC2702224

[B91] Paroni G Cernotta N Dello RC Gallinari P Pallaoro M Foti C Talamo F Orsatti L Steinkuhler C Brancolini C PP2A regulates HDAC4 nuclear import Mol Biol Cell 2008 19 655 667 18045992 10.1091/mbc.E07-06-0623 PMC2230598

[B92] Tochiki KK Cunningham J Hunt SP Geranton SM The expression of spinal methyl-CpG-binding protein 2, DNA methyltransferases and histone deacetylases is modulated in persistent pain states Mol. Pain 2012 8 14 10.1186/1744-8069-8-14 22369085 PMC3351747

[B93] Zhang Z Cai YQ Zou F Bie B Pan ZZ Epigenetic suppression of GAD65 expression mediates persistent pain Nat Med 2011 17 1448 1455 10.1038/nm.2442 21983856 PMC3210928

